# Hesitancy of Arab Healthcare Workers towards COVID-19 Vaccination: A Large-Scale Multinational Study

**DOI:** 10.3390/vaccines9050446

**Published:** 2021-05-02

**Authors:** Eyad Qunaibi, Iman Basheti, Mohamed Soudy, Iyad Sultan

**Affiliations:** 1 College of Pharmacy, Jerash Private University, Jerash 26150, Jordan; 2 Department of Clinical Pharmacy and Therapeutics, Faculty of Pharmacy, Applied Science Private University, Amman 11931, Jordan; dr_iman@asu.edu.jo; 3 Proteomics and Metabolomics Unit, Basic Research Department, Children’s Cancer Hospital, Cairo 11562, Egypt; Mohamed.soudy@57357.org; 4 Department of Paediatrics and Cancer Care Informatics Program, King Hussein Cancer Center, Amman 11941, Jordan; isultan@khcc.jo; 5 Department of Paediatrics, University of Jordan, Amman 19328, Jordan

**Keywords:** COVID-19 vaccines

## Abstract

Background: Health care workers (HCWs) are at increased risk of acquiring and transmitting COVID-19 infection. Moreover, they present role models for communities with regards to attitudes towards COVID-19 vaccination. Hence, hesitancy of HCWs towards vaccination can crucially affect the efforts aiming to contain the pandemic. Previously published studies paid little attention to HCWs in Arab countries, which have a population of over 440 million. Objectives: To assess the rates of COVID-19 vaccine hesitancy in Arabic-speaking HCWs residing in and outside Arab countries, and their perceived barriers towards vaccination. Methods: A cross-sectional study based on an online survey was conducted from 14–29 January 2021, targeting Arabic-speaking HCWs from all around the world. Results: The survey recruited 5708 eligible participants (55.6% males, 44.4% females, age 30.6 ± 10 years) from 21 Arab countries (87.5%) and 54 other countries (12.5%). Our analysis showed a significant rate of vaccine hesitancy among Arabic-speaking HCWs residing in and outside of Arab countries (25.8% and 32.8%, respectively). The highest rates of hesitancy were among participants from the western regions of the Arab world (Egypt, Morocco, Tunisia, and Algeria). The most cited reasons for hesitancy were concerns about side effects and distrust of the expedited vaccine production and healthcare policies. Factors associated with higher hesitancy included age of 30–59, previous or current suspected or confirmed COVID-19, female gender, not knowing the vaccine type authorized in the participant’s country, and not regularly receiving the influenza vaccine. Conclusion: This is the first large-scale multinational post-vaccine-availability study on COVID-19 vaccine hesitancy among HCWs. It reveals high rates of hesitancy among Arab-speaking HCWs. Unless addressed properly, this hesitancy can impede the efforts for achieving widespread vaccination and collective immunity.

## 1. Introduction

Health care workers (HCWs) are at high risk of occupational SARS-CoV-2 exposure and transmission, which prioritizes them for early COVID-19 vaccination. In addition, different communities treat HCWs as role models in their attitudes towards vaccination and refer to them for vaccine information [1]. For this reason, vaccine hesitancy among HCWs can impede the widespread implementation of vaccination and contradict the efforts for circumventing the ongoing COVID-19 pandemic; thus, assessing the size of hesitancy, in addition to addressing its root causes, is important.

A number of studies have assessed vaccine attitudes in HCWs before vaccine availability and uptake [1,2,3,4,5,6,7,8,9,10,11,12,13], with the rate of reported vaccine acceptance ranging from as low as 27.7% in Congolese HCWs [7], to as high as more than 95% in a study involving HCWs from the Asia-Pacific region [10]. Some studies found the HCWs to be more inclined to take the vaccine than non-HCWs, but still with high rates of hesitancy. [5,6]. The most common barriers against vaccination among HCWs included concerns about efficacy, safety, and the conceivably rushed vaccine production process.

In the last few months, several Arab countries have announced more coronavirus infections and fatalities, raising the total numbers from the start of the pandemic to about 4.5 million and 76 thousand, respectively, up to mid-March 2021. In a previous work [14], we showed a high rate of vaccine hesitancy in Arab countries post vaccine availability. To our knowledge, only two studies have assessed vaccine hesitancy among HCWs in Arab countries: in Saudi Arabia [3] (*n* = 673), where about a quarter intended to receive the vaccine once available; and Egypt [4] (*n* = 496), where 46% of HCWs somewhat/totally agreed to receive the vaccine. Therefore, an update of the status of vaccine hesitancy among HCWs in general, and among Arabic-speaking HCWs in particular, is required.

The aim of this study was to address vaccine attitudes among Arabic-speaking HCWs in and out of the Arab countries, after the vaccine was made available, through a large-scale online survey. The study includes a comparison with the attitudes of non-HCWs, vaccine acceptance and hesitancy indicators by demographic groups, barriers to vaccination among HCWs, and their attitudes towards vaccine-mandating policies.

## 2. Methods

A cross sectional study was conducted through an online survey to assess the attitude of Arab-speaking people toward COVID-19 vaccination. Survey data were collected from 14–29 January 2021 using the online platform www.surveyplanet.com. Unique IP addresses were only allowed to participate once, to ensure that each participant was real. Social media platforms were used to conduct a digital campaign targeting a convenience sample of Arabic-speaking HCWs from all around the world. Data were collected anonymously, and no personally identifiable information was collected or stored. Consent to participate was obtained for each participant before answering the questions of the survey. All questions were written and validated in the Arabic language. An English translation of the survey can be found in Supplementary Document 1. This study was approved by the institutional review board (IRB) at King Hussein Cancer center (Approval No. 21 KHCC 0.34).

The survey consisted of 17 questions, to capture the following data for each participant: demographics, health status, influenza vaccination, type(s) of approved COVID-19 vaccine(s) in the participant’s country, whether he/she had already received a COVID-19 vaccine and the appearance of any side effects thereof, self-perceived need for COVID-19 vaccination and mandating policies, intention of the participant to uptake the vaccine in case they did not yet, and a detailed list of barriers towards vaccination to choose from. Participants who had already taken the COVID-19 vaccine were not allowed to answer the question about the intention to take it. An English translation of the survey questions is provided in the Supplementary Materials and can be accessed online: https://tinyurl.com/5bd6j7fj.

The independent demographic variables collected, included age, gender, country of residence, academic education, and being a HCW or not. Only the data for HCWs were analyzed for the purpose of this study, while the gross data were reported elsewhere [14].

Data for age were collected through open-text fields, and age was then coded into age categories: below 30, 30–39, 40–49, 50–59, or 60 years or older. Academic education levels were categorized as lower (less than high school diploma, high school diploma, and some education with no degree) and higher (university degree or diploma and postgraduate degree).

Participants were also subcategorized according to their country of residence. Arab countries with less than 25 participants (Bahrain, Mauritania, Somalia) were grouped together in one category and labeled “Other Arab countries”. The non-Arab countries where the Arabic speaking participants resided were classified into groups: European countries (*n* = 25), North American countries (*n* = 2; the United States and Canada), Turkey, and the rest of the non-Arab countries as others (*n* = 26).

The survey data were analyzed and graphs generated using the R programming language (R Foundation for Statistical Computing, Vienna, Austria. http://www.R-project.org/ accessed on 1 March 2021) v.4.0.2. The primary outcome of the survey was whether participants were willing to take the COVID-19 vaccine or not, which was assessed b9y the question “Do you intend to take the vaccine if the option is available to you?”. The responses allowed for graded stances (Yes; Depends on the type of vaccine; No; Not sure; I will wait and see its effects on others). The first two of these choices were used to define vaccine acceptance, since those who chose ‘”depends on the type of vaccine” did not have an objection to the vaccine in principle, while the last three were labeled as “vaccine hesitancy”.

The distribution of the responses to this question was analyzed for the entire dataset and further differences by country were examined. To analyze the association between vaccine acceptance and participant characteristics, binary logistic regression was used.

Responses to the question on the barriers to acceptance were compared for gender, age, country of residence, having chronic disease, and academic education using chi-square. The acquisition and analysis of the results followed the guidelines of the CHERRIES checklist22 [15].

The COVID19 package v2.3.2 was used for updated data on COVID-19 cases and deaths. The results of the survey can be found on the project website at https://mainapp.shinyapps.io/CVHAA [16].

## 3. Results

### 3.1. Demographics

The survey recruited 6043 HCWs, who consented to participate, and of whom 335 reported that they had already taken the vaccine. Data analysis was performed for the remaining 5708 participants for the sake of this study.

The participants covered all the Arab countries and territories (*n* = 4996, 87.5%) except Djibouti and Comoros, and Arabs who lived in 54 other countries outside the Arab region (*n* = 712, 12.5%). Participants from countries outside of the Arab region were clustered into four groups: Europe (*n* = 397, 7.0%), North America (*n* = 89, 1.6%), Turkey (*n* = 168, 2.9%) and others (*n* = 58, 1.0%).

Participants from Arab countries were from Algeria, Bahrain, Egypt, Iraq, Jordan, Kuwait, Lebanon, Libya, Mauritania, Morocco, Oman, Palestine, Qatar, Saudi Arabia, Somalia, Sudan, Syria, Tunisia, UAE, and Yemen.

Participants from European countries were from the Netherlands, United Kingdom, France, Germany, Spain, Sweden, Bosnia and Herzegovina, Italy, Austria, Romania, Republic of Ireland, Switzerland, Cyprus, Ukraine, Denmark, Aruba (Netherlands), Norway, British Virgin Islands (United Kingdom), Greece, Belgium, Hungary, Czech Republic, Poland, Finland, and Luxembourg.

Participants from “other” countries were from Australia, India, Russia, Ethiopia, Japan, China, Kyrgyzstan, Cayman Islands (United Kingdom), Malaysia, Ecuador, Pakistan, Uzbekistan, Kazakhstan, Bulgaria, Belarus, South Africa, Brunei, Argentina, Georgia, Iran, Guadeloupe (France), South Korea, Chad, Jamaica, the Bahamas, and the Gambia.

The mean age of the participants was 30.6 years (±10), and more males (*n* = 3171, 55.6%) participated than females (*n* = 2537, 44.4%), Supplementary Figure S1. Chronic diseases were reported by 800 participants (14.0%). In response to the question (Do you take the flu vaccine annually), only 321 (5.6%) participants chose “yes, every year” while 3994 (70.0%) reported never receiving it. Previously confirmed or suspected COVID-19 infection was reported by 1270 (22.2%) participants; while 1859 (32.6%) participants were not sure if they had contracted the virus. From the 912 participants who reported testing for COVID-19, 337 had positive test results (37.0% positivity). Two thirds (*n* = 3875, 67.9%) of the participants had a bachelor’s degree or higher.

When asked about the type of COVID-19 vaccine authorized in their countries, 1851 (32.4%) did not know the type. More HCWs in Arab countries reported that they do not know the types of vaccines officially approved in their countries (34.3%) compared with HCWs outside of Arab countries (19.0%). Those who knew the vaccine type(s) reported authorizing the American vaccine from Pfizer–BioNTech or Moderna (*n* = 2291, 40.1%), the Chinese vaccine from Sinopharm or Sinovac (*n* = 2190, 38.4%), the European vaccine from Oxford–AstraZeneca (*n* = 623, 10.9%), the Russian Vaccine Sputnik V (*n* = 541, 9.5%), and the Indian vaccine (*n* = 55, 1%). Detailed participant characteristics are shown in Supplementary Table S1 and compared with those of the non-HCW that we reported in a previous study [14].

### 3.2. COVID-19 Vaccination Acceptance and Related Factors

**Figure 1:**
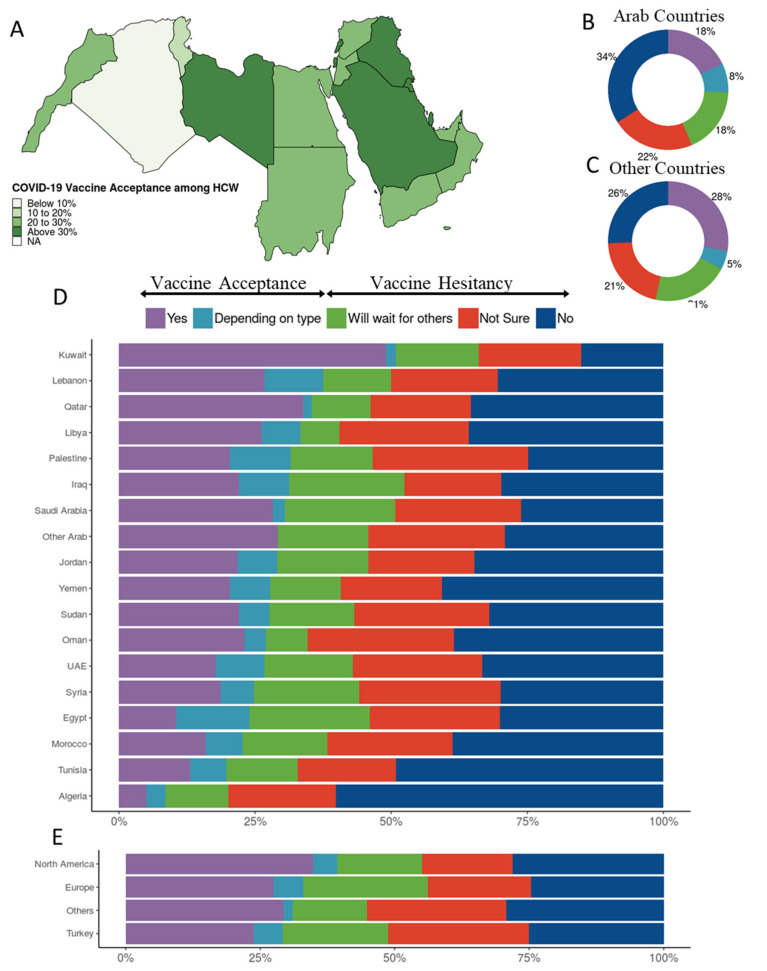
COVID-19 vaccination attitudes among 5708 HCW participants. (**A**) Vaccine acceptance per country in the Arab region, (**B**) vaccination attitudes reported by HCWs from Arab countries and territories, (**C**) vaccination attitudes reported by HCWs from countries other than Arab countries and territories, (**D**) vaccination attitudes reported by HCWs per Arab country/territory, and(**E**) vaccination attitudes reported by HCWs from countries other than Arab countries and territories clustered by residency region.

The overall COVID-19 vaccine acceptance rate in this study was 26.7%. The rate of vaccine acceptance among HCWs in Arab countries (25.8%) was lower than among their counterparts outside the Arab countries (32.8%) (Figure 1A–C, Table 1). Participants from Kuwait showed the highest vaccine acceptance (50.9%), while those from the western regions (Egypt, Morocco, Tunisia, Algeria) showed the lowest vaccine acceptance (Figure 1D). As for the HCWs residing outside the Arab region, those living in North America showed more vaccine acceptance than those in the other three clusters (Figure 1E).

**Figure 2:**
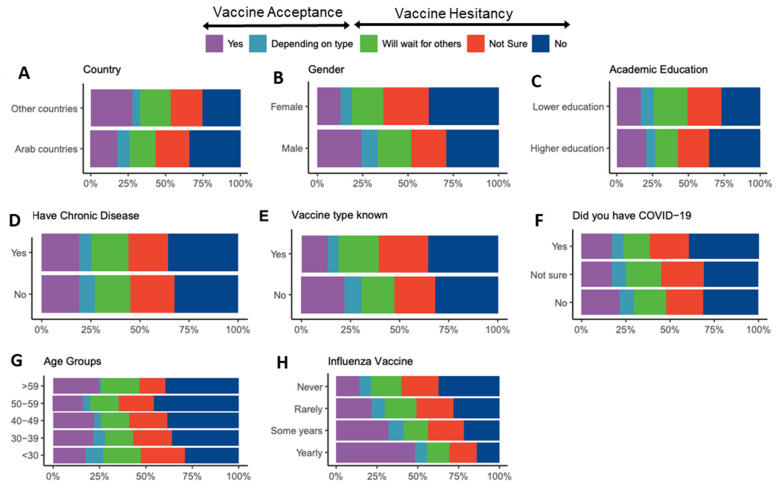
Differences in COVID-19 Vaccination attitudes among HCWs according to: (**A**) country of residence, (**B**) gender, (**C**) level of academic education, (**D**) having a chronic illness, (**E**) knowing the vaccine type available in participant’s country, (**F**) having a previous or current COVID-19 infection, (**G**) age, and (**H**) receiving annual influenza vaccine.

**Figure 3:**
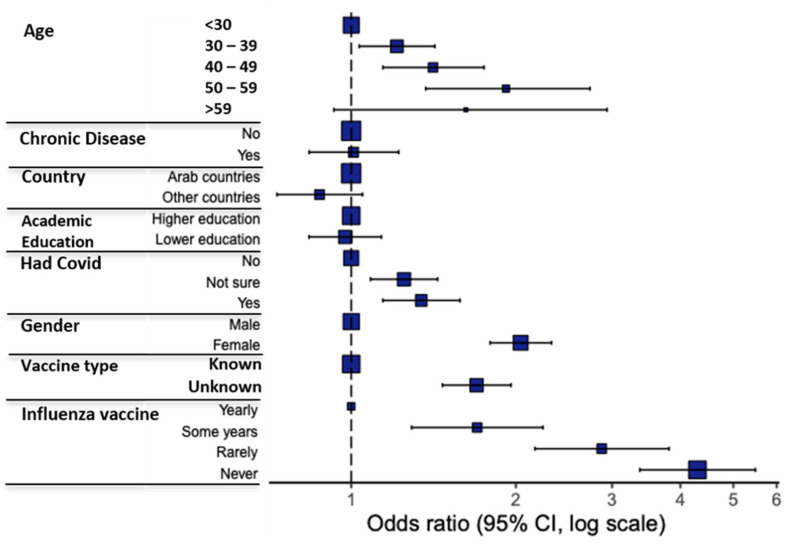
Multivariate analysis results of COVID-19 vaccine acceptance/hesitancy stratified according to different factors; odds ratio (OR) and 95% confidence intervals (CI) are shown; the size of the box represents the number of participants in each level.

Variations in response to the question of vaccine acceptance (Table 2) were analyzed using participant characteristics as covariates (Figure 2). A binomial logistic regression model was used to examine the correlation of these participant characteristics with vaccine acceptance (Figure 3, Table 3). Univariate and multivariate analyses revealed several predictors for vaccine hesitancy, with the odds ratio showing the stronger effect of the following factors on participants’ hesitance: never (OR, 4.30) or rarely (OR, 2.87) receiving the influenza vaccine, female gender (OR, 2.04), not knowing the vaccine type authorized in the participant’s country (OR, 1.69), and having or previously having suspected or confirmed COVID-19 infection (OR, 1.34).

### 3.3. Barriers to Acceptance

**Figure 4:**
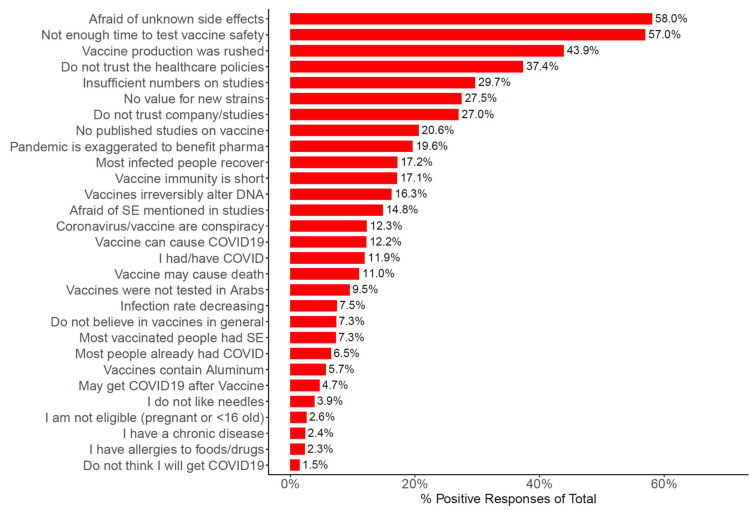
Bar plot showing percentage of participants (*n* = 5708) who selected the shown barriers.

The most common barriers among HCWs towards COVID-19 vaccination are shown in Figure 4. More than half of the participants chose “I am afraid side effects of the vaccine will develop, other than what has been disclosed” = 3313 (58.0%); “Not enough time has passed to verify the vaccine’s safety” = 3252 (57.0%); “The vaccine production has been rushed, making me doubt the credibility of the producing company” = 2505 (43.9%); and “I do not trust the healthcare policies applied in my country” = 2133 (39.1%). Most of the barriers were reported more frequently by HCWs in Arab countries compared with their counterparts outside (Supplementary Figure S2A), and by female HCWs compared with male counterparts (Supplementary Figure S2B). Notably, 41.0% of HCWs in Arab countries chose “I do not trust the healthcare policies applied in my country” versus 11.9% who chose the same barrier among their counterparts residing outside.

**Figure 5:**
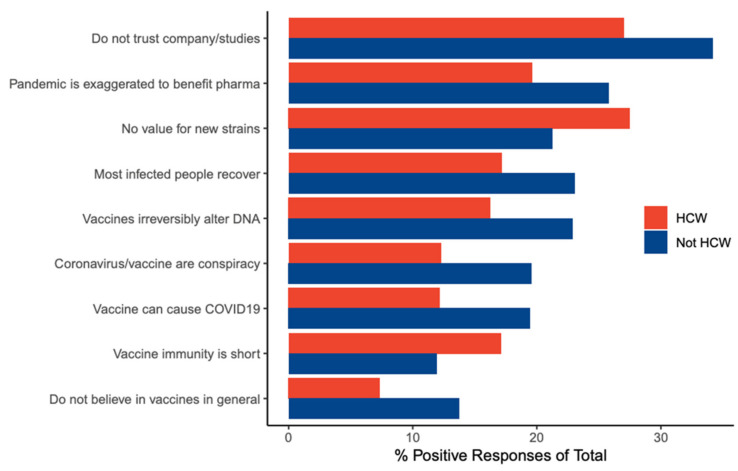
Bar plot showing the barriers with more than 5% difference between HCWs and non-HCWs.

We compared the barriers chosen by HCWs in this study with those chosen by non-HCWs that we reported in a separate study [14] (Table 4). The barriers that differ significantly (*p* < 0.001) with more than 5% difference are shown in Figure 5. Notably, the choices “The vaccine might lose its efficacy against the new viral strains” and “I believe/I fear that the immunity gained from the vaccine does not last long” were chosen more frequently by HCWs than non-HCWs.

### 3.4. Stances of HCWs on Vaccination Policies and Need

**Figure 6:**
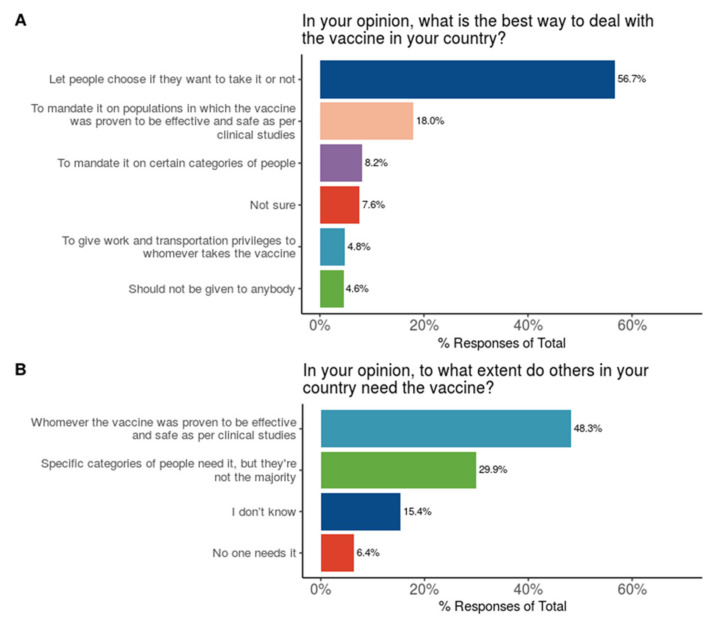
Participants’ attitudes towards COVID-19 vaccination with regards to (**A**) national health policies, and (**B**) selecting individuals who should be vaccinated.

When asked “In your opinion, what is the best way to deal with the vaccine in your country?”, more than half of the HCWs (56.7%) chose “To let people choose if they want to take it or not”. Only 16.2% of HCWs supported mandating the vaccine on some groups of people (Figure 6A). When asked “In your opinion, to what extent do others in your country need the vaccine?”, about half of the HCWs (48.3%) chose “It is needed for whomever the vaccine was proven to be effective and safe as per clinical studies” (Figure 6B).

## 4. Discussion

This study fills an important knowledge gap, being the largest multinational survey, and the first after the vaccine was made available, on the attitudes of HCWs towards COVID-19 vaccines. Only one other multinational study has been published on HCWs (*n* = 1720) from six countries, including China, India, Indonesia, Singapore, Vietnam, and Bhutan [10]. Another study (*n* = 2238) compared COVID-19 vaccine acceptance between the HCWs of two countries: Greek and Cypriot [12]. None of these countries is an Arab country. Our study covered the large geographical area of the Arab countries, which have been affected badly by the pandemic, and involved a large sample size (*n* = 5708). Comparing the answers of the HCW respondents residing in and outside the Arab countries gives an insight into the effect of cultural, socioeconomic, political, and health policy differences on their reported attitudes and barriers to vaccine acceptance.

The study shows a low rate of vaccine acceptance (26.7%) among Arab-speaking HCWs. This is consistent with previous studies on Saudi HCWs (*n* = 673), in which only about a quarter intended to have the vaccine as soon as it becomes available in their country, while another quarter would delay until the vaccine safety is confirmed [3]. Similar results were shown by Egyptian HCWs (*n* = 496), of whom 13.5% totally agreed to receive the vaccine, and 32.4% somewhat agreed [4]. In our study, 30.5% of Saudi HCWs and 24% of Egyptian HCWs were willing to receive a vaccination. These low rates of acceptance are also consistent with other studies on HCWs in the USA (*n* = 3479), in which only 36% of respondents were willing to take the vaccine as soon as it became available [1]. Similarly, in a study conducted on Los Angeles HCWs (*n* = 540), most participants (65.5%) indicated they would delay vaccination once coronavirus vaccines became available for distribution [2]. Furthermore, about a quarter of Congolese HCWs (*n* = 613) reported intent to take the vaccine [7].

The HCWs residing in the Arab countries were more hesitant than their counterparts residing outside (Figure 1B,C), with a hesitancy rate of about three quarters versus two thirds, respectively. One reason can be the significantly higher percentage of HCWs in Arab countries who do not know the types of vaccines officially approved in their countries (34.3% compared with 19.0% for HCWs outside of Arab countries). Among the barriers reported towards vaccination, distrust in healthcare policies in the country of residence seems to be an important reason for this discrepancy. The HCWs residing in the Arab countries chose this barrier about three times more than those residing outside (41.0% vs 11.9%, respectively) (Supplementary Figure S2).

Vaccine acceptance varied widely among the different Arab countries, from 8.6% among HCWs of Algeria, to 50.9% among HCWs of Kuwait (Figure 1). Again, not knowing the vaccine type (29.1% vs. 11.3% in Algeria and Kuwait, respectively), and distrust in healthcare policies in the country of residence (48.4% vs. 9.4% in Algeria and Kuwait, respectively) seem to be important contributors to this variation. In addition, this variation is in clear correlation with the number of COVID-19 cases and deaths per million in the different countries, which was as low as 2.5 thousand cases and 68 deaths per million in Algeria, and as high as 40 thousand and 232 per million in Kuwait.

The rates of vaccine acceptance in HCWs (26.7%) were higher than those for non-HCWs (15.2%), for whom we reported the rates of acceptance in a previous paper [14]. This is consistent with previous studies [5,6,11], and is expected, given the higher health, pandemic, and vaccine literacy among HCWs.

Predictors of vaccine hesitancy among HCWs included an age of 30–59, previous or current suspected or confirmed COVID-19 infection, being female, not knowing the vaccine type, and never, rarely, or intermittently receiving the influenza vaccine (as compared to regular influenza vaccination) (Figure 3 and Figure 4). Similarly, previous studies reported higher intention to vaccinate in male HCWs [1,4,8,11], HCWs of older age [11], and HCWs who received influenza vaccine during previous seasons [8].

The most frequently chosen barriers to vaccine acceptance in this study were “I am afraid side effects of the vaccine will develop, other than what has been disclosed” (58.0%), “Not enough time has passed to verify the vaccine’s safety” (57.0%), and “The vaccine production has been rushed, making me doubt the credibility of the producing company” (43.9%). This is consistent with a study on the United States HCWs (*n* = 3479), in which safety (69%), effectiveness (69%), and speed of development/approval (74%) were noted as the most common concerns regarding COVID-19 vaccination [1]. In addition, a study on Los Angeles HCWs (*n* = 540) showed that respondents were most heavily influenced by the fast-tracked development timeline (83.5%) in shaping their vaccination intent [2]. On the other hand, absence of fear over vaccine safety was associated with the likelihood of COVID-19 vaccination acceptance [10,13].

There were noticeable disparities in reported barriers to vaccination between participants in and outside of Arab countries (Supplementary Figure S2). All the top 23 barriers to vaccination were higher among HCWs in Arab countries compared with their counterparts outside.

On the other hand, several barriers were significantly more frequent among HCWs compared with non-HCWs (Figure 5). These included concerns about the vaccine effectiveness against the new viral strains and short-lived immunity, which is a likely observation given the medical and paramedical background of HCWs. However, unexpectedly, more HCWs chose “The vaccine has not been tested on a large enough number of people, just tens or hundreds”. This could be due to the fact that Arab countries were first to approve the Chinese vaccines despite the lack of affirmative data [17], which can further be affirmed by the observation that significantly more HCWs in Arab countries chose this barrier compared with HCWs outside (Supplementary Figure S2).

As for the attitudes towards mandating the vaccine, the majority of HCWs believed that the choice should be given for people to take the vaccine or not (Figure 6A). In a study on HCWs in the United States [1], 48% of the participants believed the vaccine should be voluntary and 17% were not sure. In our study, about half of the HCWs chose that the vaccine is needed for whomever it was proven to be effective and safe as per clinical studies (Figure 6B), which is about double those who intend to uptake the vaccine. This is consistent with a previous study on HCWs in Los Angeles [2], where participants overwhelmingly acknowledged the importance and utility of general vaccination to public health practice but were widely hesitant about partaking in COVID-19 vaccination in trial or post-market settings.

## 5. Conclusions

This study highlights the alarmingly high rate of COVID-19 vaccine hesitancy among HCWs after the vaccine was made available. Given the crucial role of HCWs, this is expected to significantly affect the ongoing efforts for achieving widespread vaccination and collective immunity. Concerns about vaccine safety and lack of trust in healthcare policies must be addressed by policy makers. This can be done by improving communications with HCWs using all available channels (emails, social media, webinars, etc.) and inviting them to online meetings and workshops with healthcare policy makers to listen to their concerns and suggestions. It is also crucial to have a transparent evidence-based healthcare policy and to incorporate representatives of healthcare workers in healthcare decision making. Properly addressing the problem of HCW vaccine hesitancy is crucial to support the efforts of containing the pandemic.

## Figures and Tables

**Table 1:** Acceptance and Hesitancy Rates by Country.

Country	No		Not Sure		Will Wait for Others		Depending on Type		Yes		Total
	*n*	%	*n*	%	*n*	%	*n*	%	*n*	%	
**Algeria**	287	(60.2%)	94	(19.7%)	55	(11.5%)	17	(3.6%)	24	(5.0%)	477
**Egypt**	376	(30.1%)	298	(23.8%)	276	(22.1%)	169	(13.5%)	131	(10.5%)	1250
**Europe**	98	(24.7%)	76	(19.1%)	92	(23.2%)	22	(5.5%)	109	(27.5%)	397
**Iraq**	42	(29.8%)	25	(17.7%)	30	(21.3%)	13	(9.2%)	31	(22.0%)	141
**Jordan**	379	(34.7%)	212	(19.4%)	184	(16.8%)	79	(7.2%)	238	(21.8%)	1092
**Kuwait**	8	(15.1%)	10	(18.9%)	8	(15.1%)	1	(1.9%)	26	(49.1%)	53
**Lebanon**	17	(30.4%)	11	(19.6%)	7	(12.5%)	6	(10.7%)	15	(26.8%)	56
**Libya**	15	(35.7%)	10	(23.8%)	3	(7.1%)	3	(7.1%)	11	(26.2%)	42
**Morocco**	87	(38.7%)	52	(23.1%)	35	(15.6%)	15	(6.7%)	36	(16.0%)	225
**North America**	25	(28.1%)	15	(16.9%)	14	(15.7%)	4	(4.5%)	31	(34.8%)	89
**Oman**	10	(38.5%)	7	(26.9%)	2	(7.7%)	1	(3.8%)	6	(23.1%)	26
**Other Arab**	7	(29.2%)	6	(25.0%)	4	(16.7%)	0	(0.0%)	7	(29.2%)	24
**Others**	17	(29.3%)	15	(25.9%)	8	(13.8%)	1	(1.7%)	17	(29.3%)	58
**Palestine**	66	(24.8%)	76	(28.6%)	40	(15.0%)	30	(11.3%)	54	(20.3%)	266
**Qatar**	23	(35.4%)	12	(18.5%)	7	(10.8%)	1	(1.5%)	22	(33.8%)	65
**Saudi Arabia**	142	(26.1%)	126	(23.2%)	110	(20.2%)	12	(2.2%)	154	(28.3%)	544
**Sudan**	45	(31.9%)	35	(24.8%)	22	(15.6%)	8	(5.7%)	31	(22.0%)	141
**Syria**	103	(29.9%)	89	(25.9%)	67	(19.5%)	21	(6.1%)	64	(18.6%)	344
**Tunisia**	30	(49.2%)	11	(18.0%)	8	(13.1%)	4	(6.6%)	8	(13.1%)	61
**Turkey**	42	(25.0%)	44	(26.2%)	33	(19.6%)	9	(5.4%)	40	(23.8%)	168
**UAE**	45	(33.3%)	32	(23.7%)	22	(16.3%)	12	(8.9%)	24	(17.8%)	135
**Yemen**	22	(40.7%)	10	(18.5%)	7	(13.0%)	4	(7.4%)	11	(20.4%)	54

**Table 2:** Characteristics of HCWs with Distribution of COVID-19 Vaccine Willingness.

Label	Levels	No		Not Sure		Will Wait for Others		Depending on Type		Yes	
		*n*	%	*n*	%	*n*	%	*n*	%	*n*	%
**Age**	<30	876	(28.9%)	720	(23.7%)	624	(20.6%)	290	(9.6%)	523	(17.2%)
	30–39	595	(36.1%)	345	(20.9%)	249	(15.1%)	105	(6.4%)	355	(21.5%)
	40–49	266	(38.4%)	142	(20.5%)	106	(15.3%)	26	(3.8%)	152	(22.0%)
	50–59	120	(46.0%)	49	(18.8%)	40	(15.3%)	10	(3.8%)	42	(16.1%)
	>59	29	(39.7%)	10	(13.7%)	15	(20.5%)	1	(1.4%)	18	(24.7%)
**Chronic Diseases**	No	1600	(32.6%)	1104	(22.5%)	883	(18.0%)	384	(7.8%)	937	(19.1%)
	Yes	286	(35.8%)	162	(20.3%)	151	(18.9%)	48	(6.0%)	153	(19.1%)
**Country**	Arab countries	1704	(34.1%)	1116	(22.3%)	887	(17.8%)	396	(7.9%)	893	(17.9%)
	Other countries	182	(25.6%)	150	(21.1%)	147	(20.6%)	36	(5.1%)	197	(27.7%)
**Academic Education**	Higher education	1388	(35.8%)	835	(21.5%)	607	(15.7%)	260	(6.7%)	785	(20.3%)
	Lower education	498	(27.2%)	431	(23.5%)	427	(23.3%)	172	(9.4%)	305	(16.6%)
**Had Covid**	No	809	(31.4%)	535	(20.7%)	481	(18.7%)	202	(7.8%)	552	(21.4%)
	Not sure	578	(31.1%)	451	(24.3%)	362	(19.5%)	148	(8.0%)	320	(17.2%)
	Yes	499	(39.3%)	280	(22.0%)	191	(15.0%)	82	(6.5%)	218	(17.2%)
**Job**	HCW	1886	(33.0%)	1266	(22.2%)	1034	(18.1%)	432	(7.6%)	1090	(19.1%)
**Gender**	Male	911	(28.7%)	623	(19.6%)	588	(18.5%)	285	(9.0%)	764	(24.1%)
	Female	975	(38.4%)	643	(25.3%)	446	(17.6%)	147	(5.8%)	326	(12.8%)
**Influenza Vaccine**	Yearly	45	(14.0%)	53	(16.5%)	45	(14.0%)	23	(7.2%)	155	(48.3%)
	Some years	149	(21.9%)	148	(21.7%)	105	(15.4%)	61	(9.0%)	218	(32.0%)
	Rarely	201	(28.2%)	160	(22.5%)	141	(19.8%)	57	(8.0%)	153	(21.5%)
	Never	1491	(37.3%)	905	(22.7%)	743	(18.6%)	291	(7.3%)	564	(14.1%)
**Vaccine type unknown**	No	1228	(31.8%)	800	(20.7%)	656	(17.0%)	326	(8.5%)	847	(22.0%)
	Yes	658	(35.5%)	466	(25.2%)	378	(20.4%)	106	(5.7%)	243	(13.1%)

**Table 3:** Univariate and Multivariate Analyses of Factors Affecting Hesitancy.

Variable	Levels	Acceptance (%)	Hesitancy (%)	Univariable OR (CI)	Multivariable OR (CI)
**Age**	<30	813 (26.8)	2220 (73.2)	−	−
	30–39	460 (27.9)	1189 (72.1)	0.95 (0.83–1.08)	1.21 (1.03–1.42)
	40–49	178 (25.7)	514 (74.3)	1.06 (0.88–1.28)	1.41 (1.14–1.75)
	50–59	52 (19.9)	209 (80.1)	1.47 (1.08–2.03)	1.92 (1.37–2.73)
	>59	19 (26.0)	54 (74.0)	1.04 (0.62–1.81)	1.62 (0.93–2.94)
**Chronic Diseases**	No	1321 (26.9)	3587 (73.1)	−	−
	Yes	201 (25.1)	599 (74.9)	1.10 (0.93-1.31)	1.01 (0.84–1.22)
**Country**	Arab countries	1289 (25.8)	3707 (74.2)	−	−
	Other countries	233 (32.7)	479 (67.3)	0.71 (0.60–0.85)	0.87 (0.73-1.05)
**Academic Education**	Higher education	1045 (27.0)	2830 (73.0)	−	−
	Lower education	477 (26.0)	1356 (74.0)	1.05 (0.93–1.19)	0.97 (0.84–1.14)
**Had Covid**	No	754 (29.2)	1825 (70.8)	−	−
	Not sure	468 (25.2)	1391 (74.8)	1.23 (1.07-1.41)	1.25 (1.08–1.44)
	Yes	300 (23.6)	970 (76.4)	1.34 (1.15–1.56)	1.34 (1.14-1.58)
**Gender**	Male	1049 (33.1)	2122 (66.9)	−	−
	Female	473 (18.6)	2064 (81.4)	2.16 (1.91–2.44)	2.04 (1.79–2.32)
**Vaccine Type Unknown**	No	1173 (30.4)	2684 (69.6)	−	−
	Yes	349 (18.9)	1502 (81.1)	1.88 (1.64–2.16)	1.69 (1.47–1.96)
**Influenza Vaccine**	Yearly	178 (55.5)	143 (44.5)	−	−
	Some years	279 (41.0)	402 (59.0)	1.79 (1.37–2.35)	1.70 (1.29–2.24)
	Rarely	210 (29.5)	502 (70.5)	2.98 (2.27–3.91)	2.87 (2.17–3.81)
	Never	855 (21.4)	3139 (78.6)	4.57 (3.62–5.77)	4.30 (3.37–5.49)

**Table 4:** Barriers among HCW and non-HCW participants.

**Barriers**	**HCW**		**Not HCW**		**X^2^ Statistic**	***p*** **-Value**
	*n*	(%)	*n*	(%)		
**Afraid of unknown side effects**	3313	(58.0%)	18,922	(62.0%)	20.31	<0.0001
**Not enough time to test vaccine safety**	3252	(57.0%)	16,920	(55.5%)	9.74	0.0018
**Vaccine production was rushed**	2505	(43.9%)	14,193	(46.5%)	7.99	0.0047
**Do not trust the healthcare policies**	2133	(37.4%)	12,018	(39.4%)	4.63	0.031
**Insufficient numbers of studies**	1693	(29.7%)	8208	(26.9%)	23.58	<0.0001
**No value for new strains**	1569	(27.5%)	6475	(21.2%)	120.27	<0.0001
**Do not trust company/studies**	1541	(27.0%)	10,427	(34.2%)	99.31	<0.0001
**No published studies on vaccine**	1175	(20.6%)	5911	(19.4%)	6.61	0.010
**Pandemic is exaggerated to benefit pharma**	1119	(19.6%)	7868	(25.8%)	89.01	<0.0001
**Most infected people recover**	979	(17.2%)	7036	(23.1%)	88.7	<0.0001
**Vaccine immunity is short**	976	(17.1%)	3637	(11.9%)	124.47	<0.0001
**Vaccines irreversibly alter DNA**	928	(16.3%)	6985	(22.9%)	114.28	<0.0001
**Afraid of SE mentioned in studies**	847	(14.8%)	5695	(18.7%)	42.19	<0.0001
**Coronavirus/vaccine are a conspiracy**	700	(12.3%)	5972	(19.6%)	160.99	<0.0001
**Vaccine can cause COVID19**	695	(12.2%)	5939	(19.5%)	160.83	<0.0001
**I had/have COVID**	681	(11.9%)	2875	(9.4%)	38.01	<0.0001
**Vaccine may cause death**	630	(11.0%)	4257	(14.0%)	31.15	<0.0001
**Vaccines were not tested in Arabs**	543	(9.5%)	2209	(7.2%)	38.84	<0.0001
**Infection rate decreasing**	426	(7.5%)	3056	(10.0%)	32.87	<0.0001
**Do not believe in vaccines in general**	419	(7.3%)	4186	(13.7%)	168.53	<0.0001
**Most vaccinated people had SE**	416	(7.3%)	2608	(8.5%)	8.39	0.0038
**Most people already had COVID**	373	(6.5%)	1898	(6.2%)	1.29	0.26
**Vaccines contain aluminum**	326	(5.7%)	2896	(9.5%)	80.31	<0.0001
**May get COVID19 after vaccine**	267	(4.7%)	1370	(4.5%)	0.68	0.41
**I do not like needles**	220	(3.9%)	1750	(5.7%)	30.91	<0.0001
**I am not eligible (pregnant or <16 old)**	147	(2.6%)	965	(3.2%)	4.89	0.027
**I have a chronic disease**	136	(2.4%)	1266	(4.1%)	38.39	0
**I have allergies to foods/drugs**	132	(2.3%)	716	(2.3%)	0	0.99
**I do not think I will get COVID19**	86	(1.5%)	674	(2.2%)	10.76	0.001

## Data Availability

The results of the survey can be found on the project’s website at https://mainapp.shinyapps.io/CVHAA [16].

## Supplementary Materials

The following are available online at https://www.mdpi.com/article/10.3390/vaccines9050446/s1, Supplementary Document: Survey of the Stance on COVID-19 Vaccines in Arab Countries; Figure S1: A bar plot and a pie chart showing distribution of participants according to country of residence and gender; Figure S2: Barplot comparing barriers of HCWs in Arab countries with their counterparts outside (panel A), and barriers of female HCWs with male counterparts (panel B). Table S1: Comparison between HCW and non-HCW participants.
