# Supplementary material for: Hesitancy of Arab Healthcare Workers towards COVID-19 Vaccination: A Large-Scale Multinational Study
Source: Vaccines (Basel). 2021 May 2;9(5):446. doi: 10.3390/vaccines9050446 (PMC8147447; doi:10.3390/vaccines9050446)
Supplement: Supplementary file 1 [file vaccines-09-00446-s001.zip › vaccines-1193622-supplementary.pdf]

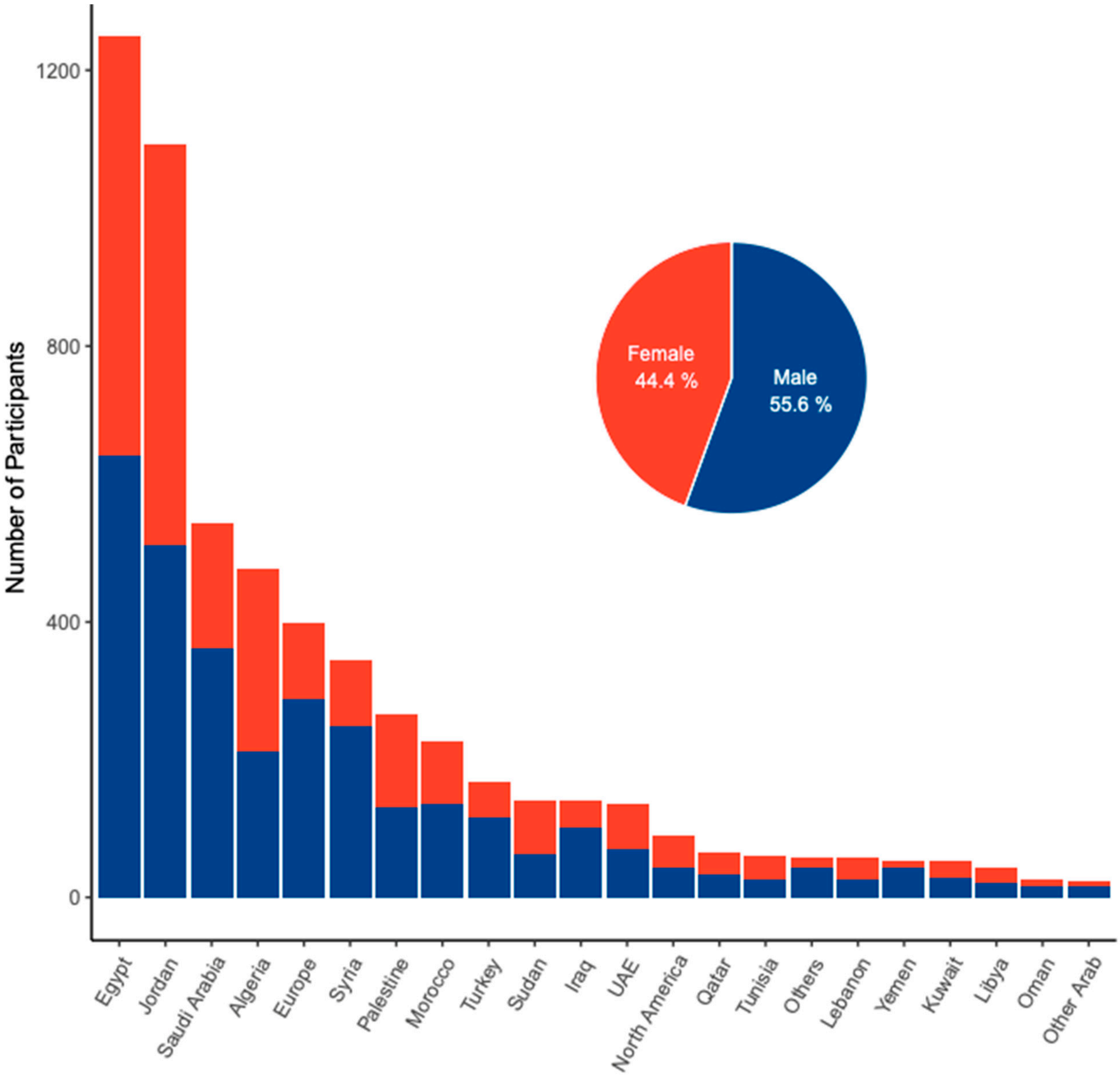

Supplementary Figure S1. A bar plot and a pie chart showing distribution of participants according to country of residence and gender.

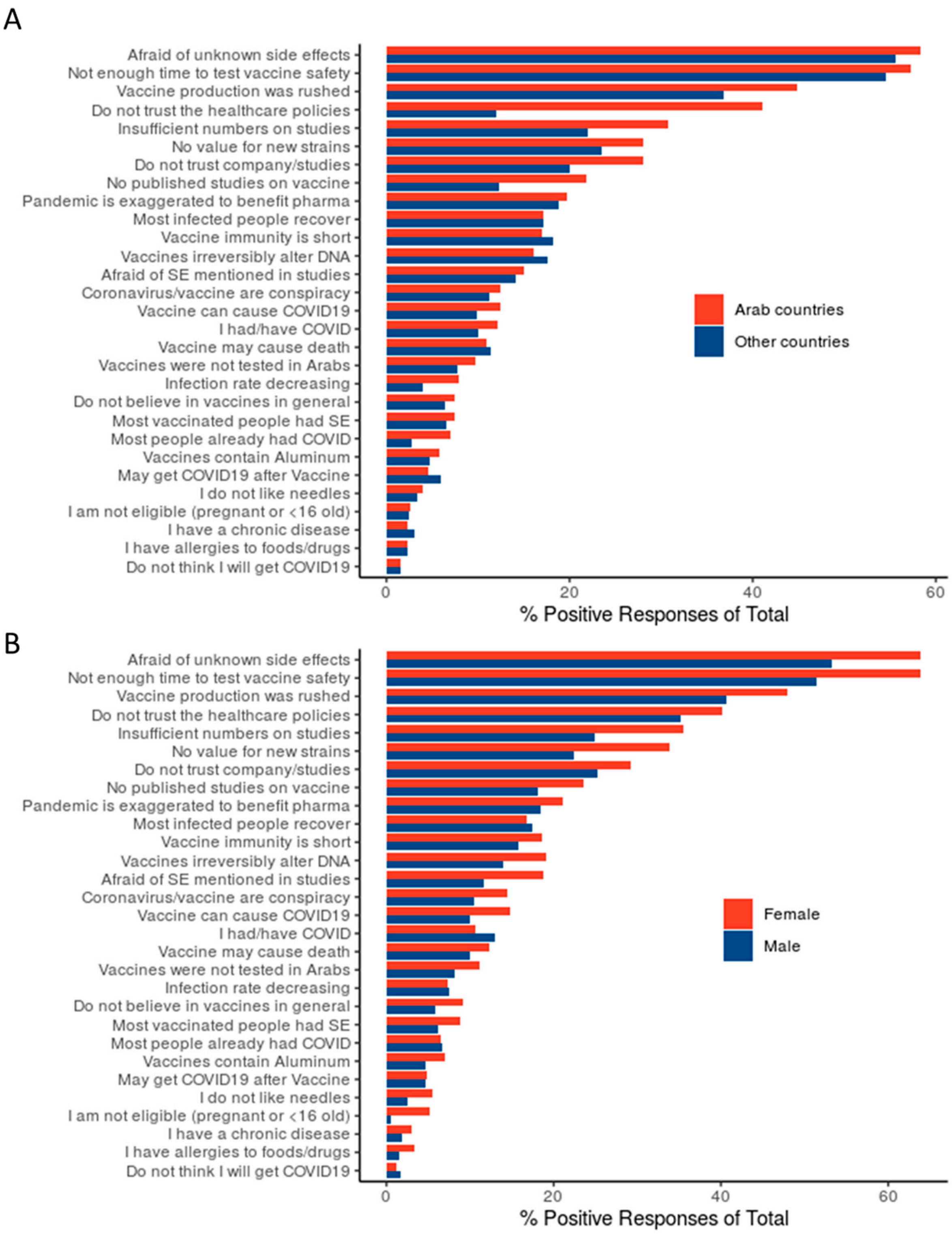

Supplementary Figure S2. Barplot comparing barriers of HCWs in Arab countries with their counterparts outside (panel A), and barriers of female HCWs with male counterparts (panel B).

Supplementary Table S1. Comparison between HCW and non-HCW participants

Label	levels	HCW		Not HCW		Total	
		N	%	N	%	N	%
**Age**	Below 30	3033	53.1%	12738	41.7%	15771	43.5%
	30-39	1649	28.9%	9916	32.5%	11565	31.9%
	40-49	692	12.1%	5227	17.1%	5919	16.3%
	50-59	261	4.6%	2040	6.7%	2301	6.4%
	Over 59	73	1.3%	591	1.9%	664	1.8%
**Chronic Diseases**	No	4908	86.0%	25473	83.5%	30381	83.9%
	Yes	800	14.0%	5039	16.5%	5839	16.1%
**Country**	Arab countries	4996	87.5%	25204	82.6%	30200	83.4%
	Other countries	712	12.5%	5308	17.4%	6020	16.6%
**Academic Education**	Higher education	3875	67.9%	18361	60.2%	22236	61.4%
	Lower education	1833	32.1%	12151	39.8%	13984	38.6%
**Had Covid**	No	2579	45.2%	15546	51.0%	18125	50.0%
	Not sure	1859	32.6%	9599	31.5%	11458	31.6%
	Yes	1270	22.2%	5367	17.6%	6637	18.3%
**Gender**	Male	3171	55.6%	18869	61.8%	22040	60.9%
	Female	2537	44.4%	11643	38.2%	14180	39.1%
**Vaccine Type Unknown**	No	3857	67.6%	17306	56.7%	21163	58.4%
	Yes	1851	32.4%	13206	43.3%	15057	41.6%
**Influenza Vaccine**	Yearly	321	5.6%	587	1.9%	908	2.5%
	Some years	681	11.9%	2230	7.3%	2911	8.0%
	Rarely	712	12.5%	3649	12.0%	4361	12.0%
	Never	3994	70.0%	24046	78.8%	28040	77.4%
**Vaccine Hesitancy**	Acceptance	1522	26.7%	4641	15.2%	6163	17.0%
	Hesitance	4186	73.3%	25871	84.8%	30057	83.0%
		5708	100.0%	30512	100.0%	36220	100.0%
